# Risk factors in self-reported dissatisfied patients implanted with various presbyopia-correcting intraocular lenses after cataract surgery

**DOI:** 10.1186/s12886-025-03912-4

**Published:** 2025-02-19

**Authors:** Woosung Jeon, Chang Ho Yoon, Joo Youn Oh, Hyuk Jin Choi, Mee Kum Kim

**Affiliations:** 1 Department of Ophthalmology, Seoul National University College of Medicine, 103 Daehak-Ro, Jongno-Gu, Seoul, 03080 South Korea; 2 Department of Ophthalmology, Seoul National University Hospital, Seoul, South Korea; 3 Laboratory of Ocular Regenerative Medicine and Immunology, Biomedical Research Institute, Seoul National University Hospital, Seoul, South Korea; 4 Department of Ophthalmology, Seoul National University Hospital Healthcare System Gangnam Center, Seoul, South Korea

**Keywords:** Dissatisfaction, Presbyopia-correcting intraocular lens, Near-vision discomfort, Photic disturbances, Preoperative myopia, Ocular aberrations

## Abstract

**Background:**

This study aimed to investigate the self-reported dissatisfaction rates and associated risk factors among patients who underwent cataract surgery using different types of presbyopia-correcting intraocular lenses (IOLs).

**Methods:**

This retrospective case–control study analyzed the medical records in 340 eyes from 211 cataract surgery patients with presbyopia-correcting IOLs. The analyzed IOL types included bifocal (ReSTOR®), trifocal (PanOptix®), and extended depth-of-focus (EDOF; Symfony®) IOLs. The rates of self-reported dissatisfaction related to vision or photic disturbances were compared between these IOLs. Various factors, including sex, age, preoperative visual acuity and refractive status, and biometric indices, were analyzed to identify potential risk factors for dissatisfaction.

**Results:**

The overall dissatisfaction rate was 18.5% (63/340). Among the IOL types, Symfony®-implanted eyes had the highest rate of near-vision dissatisfaction, while PanOptix®-implanted eyes showed similar proportions of photic disturbances and near-vision discomfort. The major risk factor identified for overall dissatisfaction, regardless of IOL type, was preoperative myopia, which aligns with the risk factor for near discomfort. Meanwhile, the risk factors for photic phenomena were revealed to be thinner corneal thickness and greater corneal astigmatism. By IOL types, preoperative myopia caused near-vision discomfort in Symfony® eyes, whereas greater corneal astigmatism and thinner corneas were linked to photic disturbances in PanOptix® eyes.

**Conclusions:**

It suggests that near-vision discomfort is related to myopic factors, whereas photic disturbances are associated with ocular aberrations. The types of dissatisfaction vary depending on the designs of presbyopia-correcting IOLs.

**Trial Registration:**

This retrospective study adhered to the principles of the Declaration of Helsinki and was approved by the Institutional Review Board of the Seoul National University Hospital on March 13, 2023 (IRB No: 2303–025-1409).

## Background

Recently, presbyopia-correcting intraocular lenses (IOLs) have gained widespread use worldwide for the correction of presbyopia [1–4]. New optical technologies have also emerged, aiming to achieve a more natural division of light. Bifocal, trifocal, and extended depth-of-focus (EDOF) IOLs are the most prominent types of IOLs [1–3]. Notably, a prior meta-analysis demonstrated that presbyopia-correcting IOLs outperformed monofocal IOLs in both intermediate and near visual acuities (VAs), despite the increased presence of halo and glare [5, 6]. Moreover, regarding the performance of different optical designs of presbyopia-correcting IOLs, the results have been presented differently depending on the study [7–12]. In general, trifocal IOLs are considered to have superior uncorrected near VA compared to EDOF IOLs, although no differences are observed in distance and intermediate VA [8, 10].

Despite these advantages, photic disturbances remain a concern with all types of presbyopia-correcting IOLs, and several studies have reported unpleasant complaints associated with these lenses [1, 5, 13–19]. Interestingly, factors such as age, postoperative refraction, and pupil size have been suggested as potential risk factors for halo in patients with presbyopia-correcting IOL implants [13, 14]. Nevertheless, there is an ongoing debate regarding the degree of patient dissatisfaction associated with different types of presbyopia-correcting IOLs, and the factors contributing to this dissatisfaction remain incompletely understood, particularly when considering the diverse designs of presbyopia-correcting IOLs.

Therefore, given the ongoing controversy surrounding the dissatisfaction trends associated with different types of presbyopia-correcting IOLs, our study aimed to investigate the clinical differences in dissatisfaction depending on the types of presbyopia-correcting IOL (EDOF, trifocal, and bifocal IOLs) among our patients. Additionally, we sought to determine whether the risk factors for dissatisfaction differ for each IOL.

## Methods

### Study design

This retrospective study adhered to the principles of the Declaration of Helsinki and was approved by the Institutional Review Board of the Seoul National University Hospital (IRB No: 2303–025–1409). Medical records of patients who underwent cataract surgery with presbyopia-correcting IOLs at Seoul National University Hospital between July 2009 and May 2022 were retrospectively reviewed. AcrySof PanOptix® (Alcon Laboratories, Inc.), Tecnis Symfony® (Johnson & Johnson Vision), or AcrySof ReSTOR® (Alcon Laboratories, Inc.) IOLs were implanted in the patients. Patients with ocular pathologies, intraoperative complications, or less than one month of follow-up were excluded from the study, resulting in a final population of 340 eyes. Data were collected regarding the type of IOL, nature of the discomfort, preoperative and postoperative VA, and other ocular parameters. Complaints were categorized as decreased distance, intermediate, or near vision, or as photic disturbances (glare, halo, or dysphotopsia).

## Outcome measures

First, we investigated the incidence of self-reported complaints based on IOL type. Second, in comparison to patients who did not report these complaints and thus constituted the control group, the risk factors were analyzed in patients who reported complaints, thereby forming the dissatisfaction group. To analyze ocular biometric parameters as potential risk factors, we evaluated postoperative satisfaction for each eye individually. Demographic information, preoperative VA, refraction, biometric and corneal topographic parameters, and postoperative VA and refraction were evaluated as risk factors. Preoperative myopia was defined as having a preoperative spherical equivalent (SE) value of less than −0.5 D. Preoperative biometric parameters (axial length; AXL, anterior chamber depth; ACD, astigmatism) were measured using IOL Master (IOL Master 500 or 700; Carl Zeiss Meditec AG) and corneal topography (ATLAS 9000; Carl Zeiss Meditec AG or Orbscan; Bausch and Lomb) devices. Central corneal thickness (CCT) and angle alpha and kappa values were measured using the IOL Master 700. Corneal spherical aberration was measured using corneal topography (ATLAS 9000; Carl Zeiss Meditec AG).

## Statistical analysis

For continuous variables, the Shapiro–Wilk test was used to test the normality of the data. Depending on the normal distribution of data, Student’s *t*-test or Mann–Whitney *U* test was used to compare the two groups. For categorical variables, the Pearson χ^2^ test or Fisher’s exact test was employed, with Bonferroni correction applied for multiple comparisons when appropriate. To analyze risk factors, the significant variables identified in the univariate analysis were subsequently included in the multivariate analysis, which was performed using generalized linear model. Statistical analyses were performed using SPSS Statistics software (v. 24.0, IBM Corp.), and the results were presented as the mean ± standard deviation (SD) and range. A *P*-value less than 0.05 was considered statistically significant.

## Results

### Baseline characteristics

**Fig. 1:**
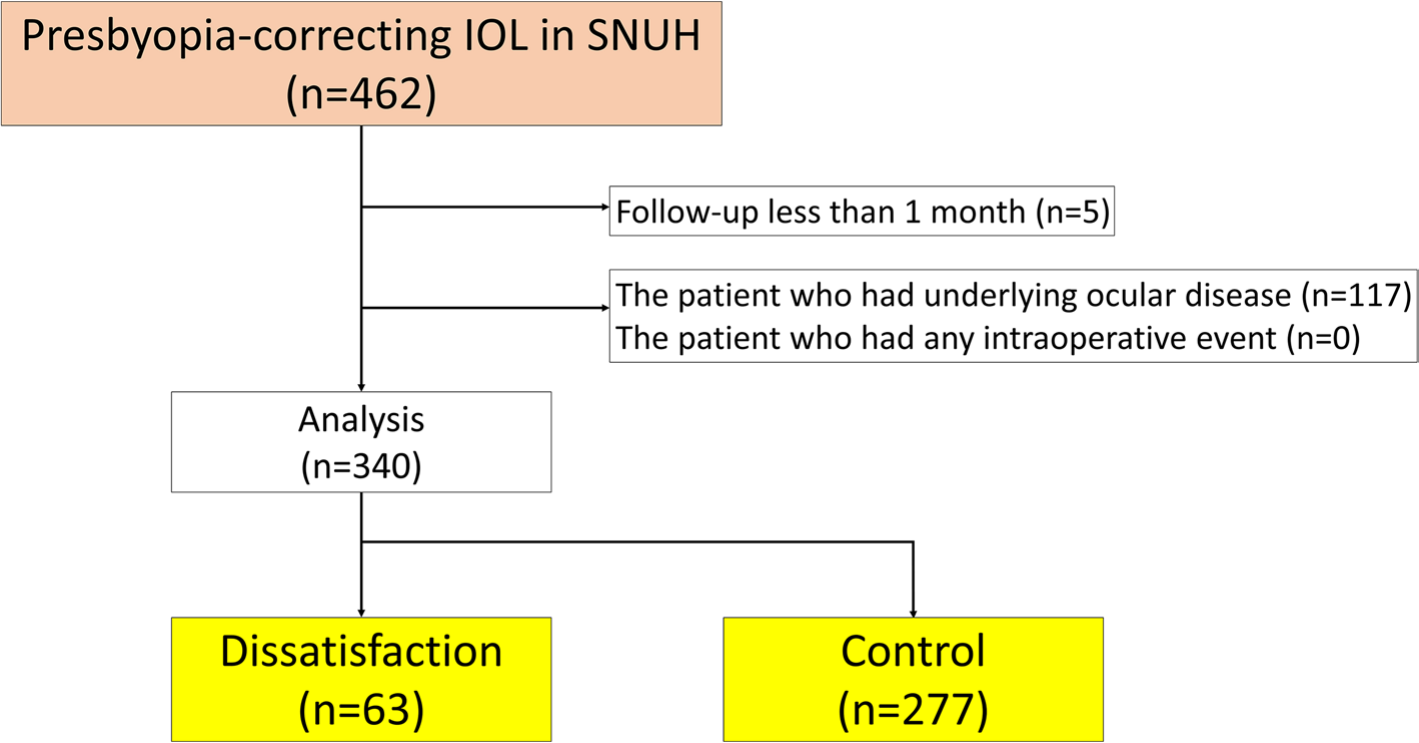
Schematic design of how to select the eyes with presbyopia-correcting IOL implantation for this study

A total of 340 eyes of 211 patients were included in the final analysis, following the exclusion of 117 eyes with underlying ocular disease and 5 eyes lost to follow-up before the 1-month post-surgery visit from an initial 462 eyes. The eyes were divided into two groups: 1) a dissatisfaction group of 63 eyes (18.5%) and 2) a control group of 277 eyes (81.5%) (Fig. 1).

Demographic factors such as age, sex, preoperative uncorrected distance VA, and ocular biometric parameters, including axial length, angle alpha, angle kappa, astigmatism, and spherical aberration, were not significantly different between the control group and the total dissatisfaction group (Table 1). Meanwhile, the postoperative distance and intermediate VA were worse in the total dissatisfaction group than in the control group (Table 2; Mann–Whitney *U* test; *P* = 0.005, *P* = 0.01), although the postoperative mean refractive error (MRE) and mean absolute refractive error (MAE) showed no significant differences (Table 2, Student’s *t*-test, *P* > 0.05).

**Table 1:** Demographics and ocular parameters of the patients with presbyopia-correcting IOL implantation

	Dissatisfaction (*n* = 63)	Control (*n* = 277)	*P*
Age (years)	60.62 ± 9.62	62.39 ± 10.52	.222^*^
Sex (Male:Female)	30:33	100:177	.090^†^
Preoperative AXL (mm)	24.17 ± 1.47	23.86 ± 1.20	.153^‡^
Preoperative astigmatism (D)	0.84 ± 0.60	0.79 ± 0.57	.510^‡^
Preoperative angle kappa	0.22 ± 0.12^a^	0.23 ± 0.12^b^	.620^*^
Preoperative angle alpha	0.42 ± 0.14^a^	0.46 ± 0.16^b^	.117^‡^
Preoperative pupil size (mm)	3.72 ± 0.56^a^	3.77 ± 0.77^c^	.670^*^
Preoperative SA	0.22 ± 0.13^d^	0.25 ± 0.12^e^	.078^*^
Preoperative UDVA (logMAR)	0.45 ± 0.31^f^	0.41 ± 0.33^ g^	.436^*^
Preoperative absolute SE	2.81 ± 3.24^ h^	2.15 ± 2.10^ g^	**.019**^**‡**^
Laterality			> .999^§^
Monocular	15	67	
Binocular	48^i^	210	
IOL type			.232^†^
PanOptix (n)	19 (16.7%)	95 (83.3%)	
Symfony (n)	28 (17.0%)	137 (83.0%)	
ReSTOR (n)	16 (26.2%)	45 (73.8%)	

AXL = axial length; D = diopter; logMAR = logarithm of the minimum angle of resolution; SA = spherical aberration; SE = spherical equivalent; UDVA = uncorrected distance visual acuity

^*^Student’s t-test, ^†^Pearson χ^2^ test, ^‡^Mann–Whitney *U* test, ^§^Fisher’s exact test

^a^50/63, ^b^240/277, ^c^241/277, ^d^47/63, ^e^208/277, ^f^56/63, ^g^197/277, ^h^51/63 eyes were analyzed in each comparison excluding missing values

^i^This included 21 patients (42 eyes) who had dissatisfaction in both eyes after surgery and 6 patients (6 eyes) who had dissatisfaction only in the one eye after surgery

**Table 2:** Visual outcomes and refractive states in dissatisfaction and control groups following presbyopia-correcting IOL implantation

	Dissatisfaction (*n* = 63)	Control (*n* = 277)	*P*
Postoperative MRE (D)	−0.24 ± 0.57	−0.31 ± 0.53	.304^*^
Postoperative MAE (D)	0.46 ± 0.41	0.49 ± 0.37	.559^*^
Postoperative UDVA (logMAR)	0.11 ± 0.17	0.04 ± 0.11^a^	**.005**^**†**^
Postoperative UIVA (logMAR)	0.18 ± 0.17^b^	0.10 ± 0.12^c^	**.014**^**†**^
Postoperative UNVA (logMAR)	0.28 ± 0.17^d^	0.23 ± 0.17^e^	.141^†^

D = diopter; logMAR = logarithm of the minimum angle of resolution; MAE = mean absolute refractive error; MRE = mean refractive error; UDVA = uncorrected distance visual acuity; UIVA = uncorrected intermediate visual acuity; UNVA = uncorrected near visual acuity

^*^Student’s t-test, ^†^Mann–Whitney *U* test

^a^268/277, ^b^33/63, ^c^171/277, ^d^41/63, ^e^181/277 eyes were analyzed in each comparison excluding missing values

## Overall incidence of dissatisfaction

**Fig. 2:**
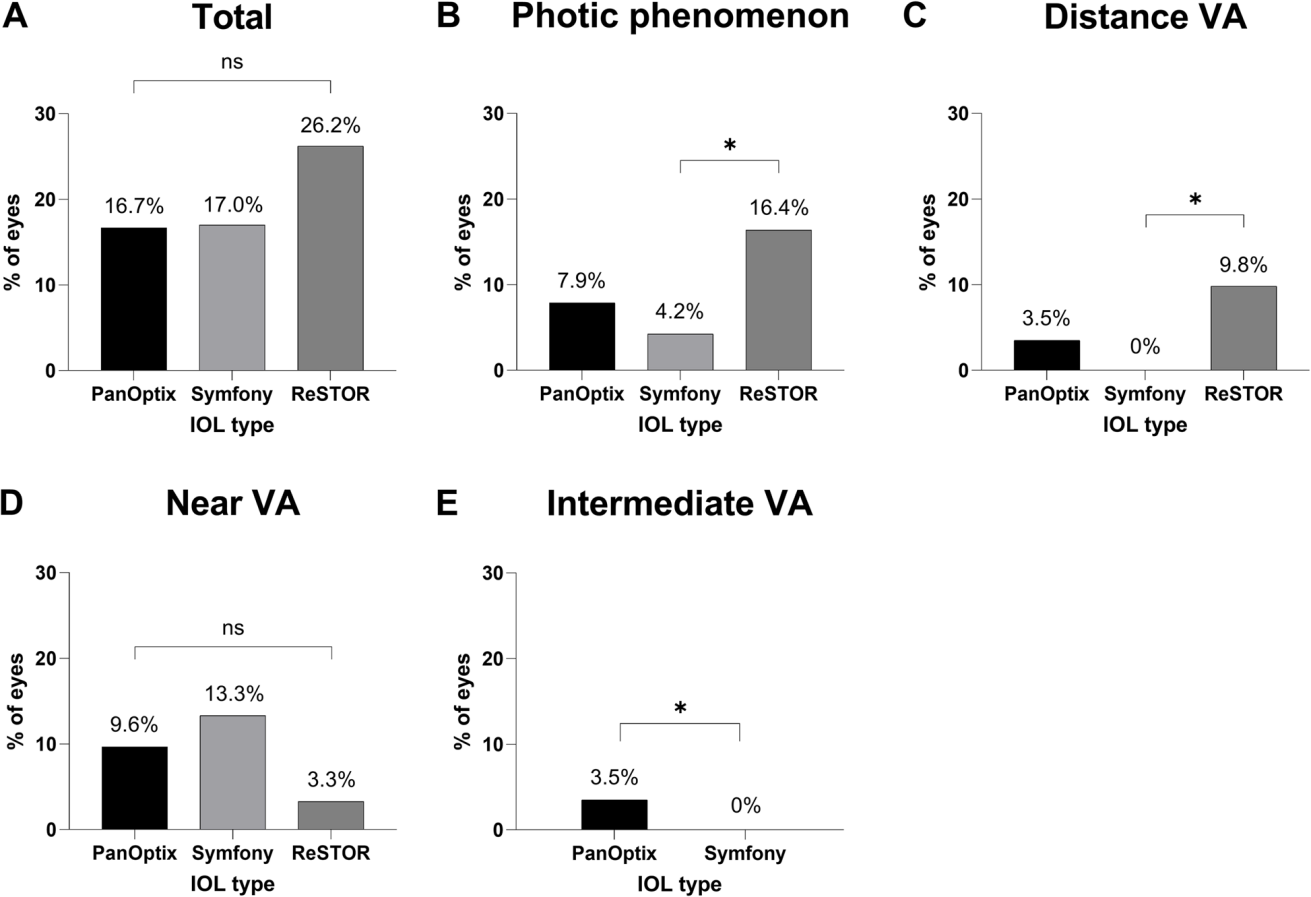
Various patterns of dissatisfaction are depicted based on the types of IOLs. **A** The proportion of dissatisfaction for each IOL type regardless of the nature of dissatisfaction. **B** The proportion of dissatisfaction of photic phenomena according to IOL types, with the ReSTOR® group having the highest rate of 16.4%, which was significantly different from the Symfony® group. **C** The proportion of dissatisfaction of distance visual acuity according to IOL types, with the ReSTOR® group having the highest rate of 9.8%, which was significantly different from the Symfony® group. **D** The proportion of dissatisfaction of near visual acuity according to IOL types, with no significant differences observed. **E** The proportion of dissatisfaction of intermediate visual acuity according to IOL types, with the PanOptix® group having the rate of 3.5%, while the Symfony® group had a rate of 0%

**Fig. 3:**
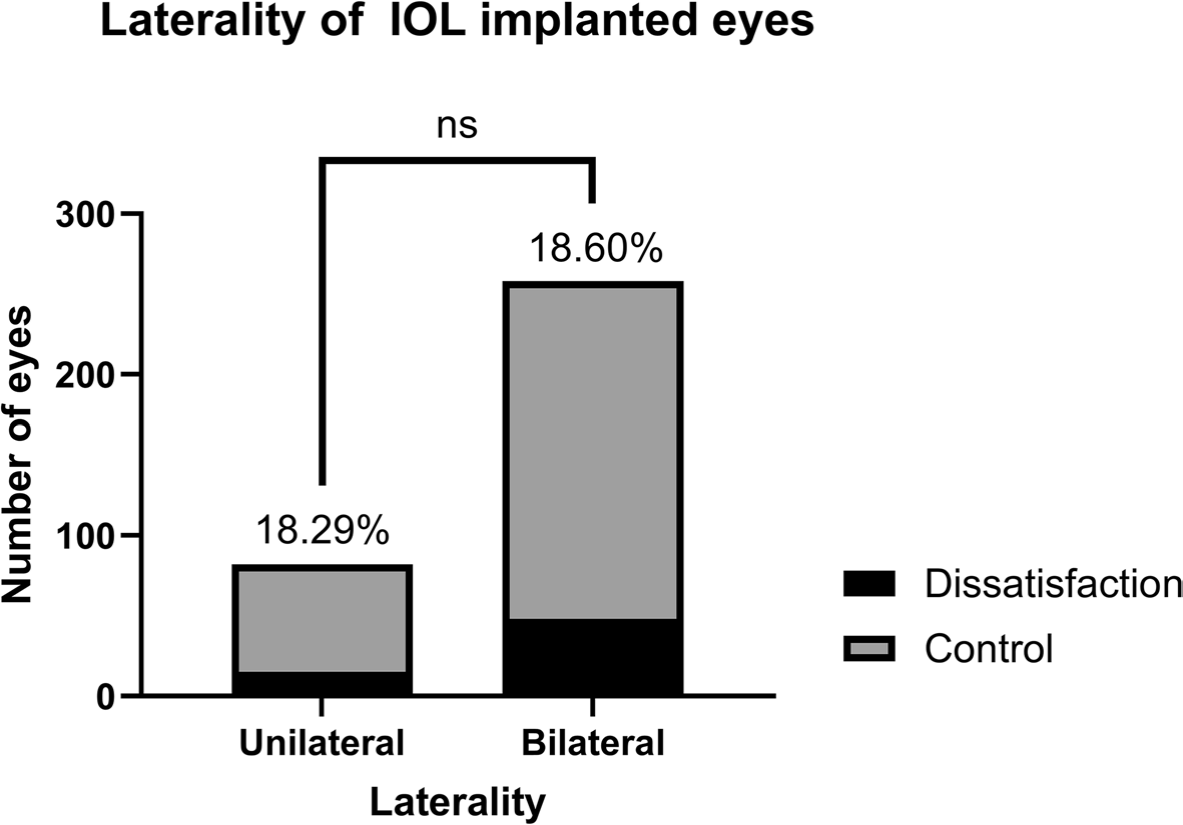
The proportion of dissatisfaction according to the laterality of IOL implanted eyes

The overall incidence of dissatisfaction after presbyopia-correcting IOL implantation was 18.5% (63/340). Examining dissatisfaction incidences in each subgroup revealed rates of 16.7% (19/114) with AcrySof PanOptix®, 17.0% (28/165) with Tecnis Symfony®, and 26.2% (16/61) with AcrySof ReSTOR® implantation, which were not significantly different (Table 1, Fig. 2; Pearson χ^2^ test; *P* = 0.23). Furthermore, there was no significant difference in the incidence of dissatisfaction between the group that underwent surgery in both eyes (48/258, 18.60%) and the group that underwent surgery in one eye (15/82, 18.29%). (Table 1, Fig. 3; Fisher’s exact test; P > 0.05).

Upon closer examination of the patients who underwent unilateral surgery, a total of 15 dissatisfied eyes were identified. Of these, 11 had contralateral eyes that were not operated on, and 4 had contralateral eyes implanted with monofocal IOLs. Among the 67 non-dissatisfied eyes, 52 had contralateral eyes that were not operated on, 10 had monofocal IOLs, 3 had presbyopia-correcting IOLs, and 2 had IOLs of unknown types.

## Incidence of dissatisfaction by type

The incidence of the categorized complaints was analyzed based on the presbyopia-correcting IOL type, as shown in Fig. 2. Near-vision dissatisfaction was the most common in the Tecnis Symfony® group; however, this result was not significant. In contrast, the ReSTOR® group exhibited significantly elevated incidences of complaints associated with photic disturbances (16.4%) and disturbed far vision (9.8%) compared to the Tecnis Symfony® group (4.2%, 0%) (Fig. 2; Fisher’s exact test; *P* = 0.01, *P* < 0.001). Additionally, the discomfort incidence of intermediate vision was higher (3.5%) in the AcrySof PanOptix® group than in the Tecnis Symfony® group (0%) (Fig. 2, Fisher’s exact test, *P* = 0.03). Therefore, complaints of photic disturbances and decreased far vision were the most common after bifocal IOL implantation.

## Risk factors contributing to dissatisfaction

When all IOL types were analyzed together, preoperative myopic proportion (< −0.5 D) and age younger than sixty (< 60) were significantly higher in the total dissatisfaction group than in the control group (Table 3; Pearson χ^2^ test; *P* < 0.001, *P* = 0.02) regardless of the type of complaints. Among them, preoperative myopia was identified as a significant risk factor, increasing the likelihood of dissatisfaction with a regression coefficient of approximately 1.080 (Table 3, Generalized linear model, *P* = 0.001). Subsequently, we evaluated whether the risk factors differed based on the type of discomfort. Notably, patients with dissatisfaction associated with photic disturbances had thinner corneas and higher astigmatism (Table 3; ^*^Student’s *t*-test, ^†^Mann–Whitney *U* test; *P* = 0.007^*^, *P* = 0.01^†^) with a regression coefficient of −0.037 and 0.886, respectively (Table 3; Generalized linear model; *P* < 0.001, *P* = 0.04). Additionally, regarding near vision, factors such as preoperative myopic proportion (< −0.5 D), age under 60, male sex, longer axial length, deeper anterior chamber, and flatter keratometric value were significantly related to dissatisfaction, irrespective of presbyopia-correcting IOL types (Table 3; ^*^Pearson χ^2^ test, ^†^Mann–Whitney *U* test, ^‡^Student’s *t*-test; *P* < 0.001^*^, *P* = 0.01^*^, *P* = 0.04^*^, *P* < 0.001^†^, *P* = 0.007^‡^, *P* = 0.04^‡^). Nonetheless, among these, preoperative myopia alone was found to significantly increase the likelihood of dissatisfaction, with a regression coefficient of 2.228 (Table 3, Generalized linear model, *P* < 0.001).

**Table 3:** Risk Factors contributing to dissatisfaction after presbyopia-correcting IOL implantation

	Dissatisfaction	Control	Univariate	Multivariate	
			*P*	B	*P*
**Total**	*n* = 63	*n* = 277			
Preoperative myopia (< −0.5D: ≥ −0.5D)^a^	26:25^a^	48:149^b^	< .001^*^	**1.080**	**.001**^**†**^
Age (< 60: ≥ 60)	28:35	81:196	.020^*^		NS
**Photic phenomenon**	*n* = 26	*n* = 314			
CCT (mm)	506.01 ± 47.82^c^	540.54 ± 30.76^d^	**.007**^**‡**^	**−0.037**	** < .001**^**†**^
Preoperative corneal astigmatism (D)	1.03 ± 0.61^c^	0.73 ± 0.56^e^	**.011**^**§**^	**0.886**	**.036**^**†**^
**Near discomfort**	*n* = 35	*n* = 305			
Preoperative myopia (< −0.5D: ≥ −0.5D)	19:6^f^	55:168^ g^	< .001^*^	**2.228**	** < .001**^**†**^
Age (< 60: ≥ 60)	18:17	91:214	.010^*^		NS
Sex (Male:Female)	19:16	111:194	.039^*^		NS
AXL (mm)	24.70 ± 1.58	23.83 ± 1.19	** < .001**^**§**^		NS
ACD (mm)	3.36 ± 0.46	3.15 ± 0.43^ h^	**.007**^**‡**^		NS
Average K (D)	43.46 ± 1.72	44.00 ± 1.46	**.044**^**‡**^		NS

B = Regression coefficient; NS = Not significant

ACD = anterior chamber depth; AXL = axial length; CCT = central corneal thickness; D = diopter; K = keratometry value

^*^Pearson χ^2^test,^†^Generalized linear model, ^‡^Student’s t-test,^§^Mann–Whitney *U* test

^a^51/63,^b^197/277,^c^18/26,^d^272/314,^e^237/314, ^f^25/35,^g^223/305,^h^291/305 eyes were analyzed in each comparison excluding missing values

After conducting a comparative analysis of various demographic and ocular factors between the two groups, considering both the type of complaints and the type of presbyopia-correcting IOL, the factors showing significant differences are presented in Table 4. Concerning presbyopia-correcting IOL types, in the PanOptix® group, thinner corneal thickness and higher astigmatism were significantly associated with photic complaints (Table 4; ^*^Student’s *t*-test, ^†^Mann–Whitney U test; *P* = 0.05^*^, *P* = 0.03^†^), with a regression coefficient of −0.047 and 1.271, respectively (Table 4; Generalized linear model; *P* = 0.007, *P* = 0.03). Whereas higher spherical aberration value and larger pupil size were significantly associated with photic complaints with ReSTOR® (Table 4; Student’s *t*-test; *P* = 0.001, *P* = 0.02). However, these factors were not identified as definitive risk factors for dissatisfaction.

**Table 4:** Risk Factors contributing to dissatisfaction after presbyopia-correcting IOL implantation depending on IOL subtypes

**PanOptix**	Dissatisfaction	Control	Univariate	Multivariate	
			*P*	B	*P*
**Total**	*n* = 19	*n* = 95			
Preoperative myopia (< −0.5D: ≥ −0.5D)	9:5^a^	15:53^b^	.003^*^	**1.775**	**.047**^**†**^
Sex (Male:Female)	9:10	20:75	.023^*^	**2.160**	**.027**^**†**^
AXL (mm)	24.76 ± 1.75	23.72 ± 1.07	**.021**^**‡**^		NS
CCT (mm)	519.16 ± 38.93	541.18 ± 31.39	**.030**^**‡**^	**−0.034**	**.018**^**†**^
**Photic**	*n* = 9	*n* = 105			
CCT (mm)	505.44 ± 44.95	540.26 ± 31.22	**.050**^**‡**^	**−0.047**	**.007**^**†**^
WTW (mm)	12.05 ± 0.42	11.75 ± 0.37	**.021**^**‡**^		NS
Preoperative corneal astigmatism (D)	1.21 ± 0.71	0.75 ± 0.59^c^	**.030**^**§**^	**1.271**	**.029**^**†**^
**Near**	*n* = 11	*n* = 103			
Preoperative myopia (< −0.5D: ≥ −0.5D)	7:0^d^	17:58^e^	< .001^*^		NS
Sex (Male:Female)	7:4	22:81	.006^*^		NS
AXL (mm)	25.62 ± 1.75	23.71 ± 1.06	**.005**^**‡**^		NS
Average K (D)	41.97 ± 1.88	44.04 ± 1.10	** < .001**^**‡**^		NS
**Symfony**	Dissatisfaction	Control	Univariate	Multivariate	
			*P*	B	*P*
**Total**	*n* = 28	*n* = 137			
Preoperative myopia (< −0.5D: ≥ −0.5D)	14:9^f^	25:70^ g^	**.002**^¶^	**1.471**	**.002**^**†**^
**Near**	*n* = 22	*n* = 143			
Preoperative myopia (< −0.5D: ≥ −0.5D)	12:5^ h^	27:74^i^	** < .001**^¶^	**1.430**	**.023**^**†**^
Age (< 60: ≥ 60)	14:8	47:96	**.005**^¶^		NS
ACD (mm)	3.41 ± 0.46	3.19 ± 0.43	**.026**^**‡**^		NS
**ReSTOR**	Dissatisfaction	Control	Univariate	Multivariate	
			*P*	B	*P*
**Total**	*n* = 16	*n* = 45			
Preoperative angle kappa	5.51 ± 0.55^j^	4.92 ± 1.19^ k^	**.013**^**‡**^		NS
**Photic**	*n* = 10	*n* = 51			
Preoperative SA	0.34 ± 0.20^ l^	0.25 ± 0.10^ m^	**.001**^**‡**^		NS
Preoperative pupil size (mm)	5.98 ± 0.17^ l^	5.36 ± 1.04^n^	**.022**^**‡**^		NS

B = Regression coefficient; NS = Not significant

ACD = anterior chamber depth; AXL = axial length; CCT = central corneal thickness; D = diopter; K = keratometry value; SA = spherical aberration; WTW = white to white

^*^Fisher’s exact test, ^†^Generalized linear model, ^‡^Student’s t-test, ^§^Mann–Whitney *U* test, ^¶^Pearson χ^2^ test

^a^14/19, ^b^68/95, ^c^99/105, ^d^7/11, ^e^75/103, ^f^23/28, ^g^95/137, ^h^17/22, ^i^101/143, ^j^15/16, ^k^43/45, ^l^4/10, ^m^23/51, ^n^19/51 eyes were analyzed in each comparison excluding missing values

None of the variables showed statistical significance for photic dissatisfaction in Symfony® eyes, nor for near dissatisfaction in ReSTOR® eyes (data not shown)

In the context of near visual discomforts, preoperative myopia (< −0.5 D), male sex, longer axial length, and flatter keratometric value were significantly related to discomfort in the PanOptix® group. However, none of these variables emerged as definitive risk factors for dissatisfaction by generalized linear model (Table 4; ^*^Fisher’s exact test, ^†^Student’s *t*-test; *P* < 0.001^*^, *P* = 0.006^*^, *P* = 0.005^†^, *P* < 0.001^†^). In the Tecnis Symfony® group, preoperative myopia (< −0.5 D) was significantly associated with near-vision discomfort (Table 4, Pearson χ^2^ test, *P* < 0.001), with a regression coefficient of 1.430 (Table 4, Generalized linear model, *P* = 0.02).

## Discussion

This study is noteworthy as it revealed distinct types of self-reported complaints based on presbyopia-correcting IOL types and identified different risk factors depending on both discomfort subtypes and types of presbyopia-correcting IOLs. We found that the implantation of either trifocal or EDOF IOLs tends to result in less dissatisfaction compared to bifocal IOL implantation, particularly in terms of photic phenomena and far vision discomfort. Interestingly, photic disturbances seem to be more related to optical factors, whereas near-vision dissatisfaction appears to be more related to myopic factors, male sex, and younger age, aligning partially with findings from prior studies [3, 13, 14].

Previous studies have compared the clinical outcomes of various presbyopia-correcting IOLs, including PanOptix®, ReSTOR®, and Symfony®. Among them, one study used VFQ-25 scores (0 to 100, higher scores indicate better satisfaction) to evaluate patient satisfaction. For photic phenomena, PanOptix® scored lower (51) than Symfony® (61.5), indicating greater dissatisfaction with glare and halo for PanOptix®. However, PanOptix® demonstrated better scores for far and near vision (88.3 and 86.6) compared to Symfony® (77.4 and 65.5) [20].

In addition to this, various other studies reporting dissatisfaction rates are summarized in Table 5 [21–29]. A review of these studies reveals that, similar to our findings, patients with Symfony® frequently reported near discomfort, while those with PanOptix® tended to experience photic phenomena [20, 21, 24]. Conversely, in the case of ReSTOR®, unlike our findings, near discomfort was reported nearly as frequently as photic phenomena in several studies [23, 25, 26, 28]. This discrepancy may be attributable to the relatively smaller number of participants in our study. Additionally, it is worth noting that certain studies did not clearly disclose postoperative SE values, which may also contribute to the observed differences.

**Table 5:** Literature review of postoperative visual outcomes and dissatisfaction rates following cataract surgery with presbyopia-correcting IOLs

	Study type	Number of eyes	IOL type	SE	UDVA (LogMAR)	UIVA (LogMAR)	UNVA (LogMAR)	Photic	Far discomfort	Near discomfort
Jeon et al.^*^	R	114	PanOptix	0.08 ± 0.41	0.07 ± 0.13	0.10 ± 0.11	0.18 ± 0.15	7.9%	3.5%	9.6%
		165	Symfony	−0.67 ± 0.38	0.03 ± 0.12	0.12 ± 0.15	0.31 ± 0.16	4.2%	0%	13.3%
		61	ReSTOR	0.00 ± 0.44	0.10 ± 0.12	0.25 ± 0.14	0.17 ± 0.18	16.4%	9.8%	3.3%
Boris M et al. (2024)	R	32	PanOptix	0.12 ± 0.58	0.09 ± 0.10	0.23 ± 0.14	0.09 ± 0.11	19.3%^a^	NA	4.5%^b^
		32	AT LISA	−0.48 ± 0.58	0.15 ± 0.16	0.31 ± 0.17	0.15 ± 0.12	21.6%^a^	NA	9.1%^b^
Galvis V et al. (2022)	R	130	PanOptix	−0.14 ± 0.33	0.04 ± 0.06	0.07 ± 0.08	0.05 ± 0.08	NA	NA	12.1%^c^
Hovanesian JA et al. (2021)	P	118	PanOptix	NA	NA	NA	NA	30%^d^	0%^e^	17%^c^
		204	ReSTOR 2.5 mini-mono	NA	NA	NA	NA	26%^d^	6%^e^	69%^c^
		178	ReSTOR 2.5/3.0	NA	NA	NA	NA	29%^d^	10%^e^	60%^c^
Lubinski W et al. (2020)	P	40	AT LISA	−0.30 ± 0.22	−0.12 ± 0.1	−0.01 ± 0.04	−0.01 ± 0.04	20%^f^	NA	0%
		40	Symfony	−0.45 ± 0.36	0.08 ± 0.08	0.09 ± 0.09	0.21 ± 0.15	5%^f^	NA	10%^c^
Hovanesian JA et al. (2019)	P	156	ReSTOR 3.0/3.0	NA	NA	NA	NA	57%^d^	12%^e^	59%^c^
		204	ReSTOR 2.5 mini-mono	NA	NA	NA	NA	26%^d^	6%^e^	68%^c^
		178	ReSTOR 2.5/3.0	NA	NA	NA	NA	29%^d^	10%^e^	60%^c^
Hovanesian JA (2018)	P	136	Crystalens	NA	NA	NA	NA	31%^f^	30%^e^	52%^c^
		98	ReSTOR or Tecnis multifocal 4.0D	NA	NA	NA	NA	56%^f^	25%^e^	37%^c^
Maxwell A et al. (2017)	P	310	ReSTOR 2.5D	NA	NA	0.34 / 0.33^ g^	0.55	25%^h^	NA	NA
Maurino V et al. (2015)	P	168	AT LISA	0.00	−0.03	0.10	0.07	27.0%^i^	4.8%^e^	10.7%^c^
		168	ReSTOR	0.13	−0.03	0.13	0.09	27.4%^i^	4.8%^e^	21.5%^c^
Lane SS et al. (2010)	P	294	ReSTOR 3.0D	0.0 ± 0.4 / 0.1 ± 0.4	0.04 ± 0.13	0.17 ± 0.14	0.10 ± 0.16	NA	NA	NA

SE = Spherical equivalent; UDVA = uncorrected distance visual acuity; UIVA = uncorrected intermediate visual acuity; UNVA = uncorrected near visual acuity; logMAR = logarithm of the minimum angle of resolution; P = Prospective; R = Retrospective; NA = Not available

^*^Results of the present study

Includes reports of “^a^glare/halo/flashes/flare, ^b^spectacles use, ^c^need glasses for near tasks, ^d^glare and halo (a fair amount to extremely), ^e^need glasses for distance tasks, ^f^glare/halo, ^h^glare/ halo/starbursts (often to always), and ^I^glare/halo/starbursts (a little bothersome to very bothersome)”

^g^Results of “the first eye / the second eye”

Photic phenomena, such as halo, glare, starburst, and dysphotopsia, are common causes of dissatisfaction in patients with presbyopia-correcting IOL implants [30]. These phenomena occur when a focused image overlaps with one or more out-of-focus images generated at different magnifications within the illuminated optical zone of the presbyopia-correcting IOL [31]. Notably, this phenomenon intensifies as either the near-add power or the light distribution near the IOL increases. In the present study, the bifocal group showed a significantly higher incidence of photic discomfort than the trifocal and EDOF groups. This may be attributed to the ReSTOR® IOL, which has a higher near-energy distribution than the other two IOLs, potentially contributing to the increased manifestation of such symptoms.

Higher-order aberrations (HOAs) and corneal astigmatism are also associated with photic phenomena after presbyopia-correcting IOL insertion [32–34]. In this study, higher degrees of corneal astigmatism were associated with photic phenomena in all patients analyzed. Interestingly, patients who were dissatisfied with the photic phenomenon had a significantly lower CCT. This finding is of significance since a thinner CCT is associated with increased total HOAs and spherical aberration [35, 36]. Moreover, Wang et al. reported a significant increase in HOAs in the cornea after cataract surgery in patients with a thinner CCT before surgery. [37] These factors suggest that a thinner CCT may play a role in the exacerbation of the photic phenomenon. Notably, wider pupil sizes are related to higher HOA values and induce photic phenomena [38, 39], and when considering only the ReSTOR® group, larger pupil sizes were indeed found to be associated with these phenomena. Therefore, second-order ocular aberrations may contribute to the photic disturbances in this study.

Given the low tolerability of presbyopia-correcting IOLs to minimal ocular aberrations, thorough preoperative counseling regarding the limitations of presbyopia-correcting IOLs and proper selection of patients are critical to achieving maximal satisfaction after implantation [3]. However, despite detailed counseling and careful patient selection, we observed that 13% of patients with EDOF IOLs and 10% of patients with trifocal IOLs reported dissatisfaction with their near vision. While it is well-established that near vision with EDOF IOLs is not as good as that with trifocal or bifocal IOLs [8–10], in this study, the incidence of near visual dissatisfaction was not statistically significant between the EDOF- and trifocal IOL-implanted patients. Consequently, preoperative myopia and related biometric factors (longer AXL, flatter keratometric values, and deeper ACD) appear to be crucial in near vision-dissatisfied patients for both types of presbyopia-correcting IOLs. Therefore, it is plausible that myopic patients tend to demand better near vision and may be disappointed more easily than hyperopic or emmetropic patients, a tendency not significantly different depending on the IOL subtype. Ultimately, it is important to provide proper warnings about near-vision dissatisfaction to patients with preoperative myopia (< −0.5 D) and the other related factors.

Interestingly, younger age and male sex were related to dissatisfaction with near vision with either the EDOF or trifocal IOLs, even though these factors were not identified as statistically significant risk factors. Thus, this suggests that younger individuals or males tend to demand work-related fine near vision, and the near vision provided by the presbyopia-correcting IOL may not be satisfactory enough for them. Given that younger age is also related to more complaints of halo and glare with both trifocal and EDOF IOLs [14], young individuals with preoperative myopia may not be suitable candidates for presbyopia-correcting IOLs because of the risk of near-vision dissatisfaction. Furthermore, the postoperative far and intermediate vision was worse in the dissatisfied group than in the control group, although the preoperative parameters and postoperative MRE or MAE values were not different. Therefore, this aspect warrants further investigation to determine its underlying causes.

This study has several limitations. First, the main outcomes of dissatisfaction were subjective, making it challenging to obtain precise results. However, the primary aim of this study was to analyze the risk factors related to self-reported complaints in real-world. Thus, it is essential to acknowledge the elements associated with patient dissatisfaction. Second, in this study, the lenticular myopia may be included in some portion because the refractive sate of preoperative myopia dose not differentiate the non-lenticular myopia from the lenticular myopia. However, other myopic factors such as longer axial length, deeper anterior chamber, and flattened cornea indicate that state of the myopia seems to be related to non-lenticular myopia. Even if some lenticular myopic patients were included, it did not interfere with the original purpose of this study. We believe that preoperative myopia, regardless of its types, can affect postoperative self-reported near-vision dissatisfaction. It is possible that patients accustomed to reading comfortably due to lenticular myopia before surgery may experience dissatisfaction with near vision post-surgery. Third, the study covered only three types of presbyopia-correcting IOLs, leaving room for further research on other types of IOLs. Additionally, the study subjects were chosen from patients who underwent surgery by four different surgeons, which could raise concerns about confounding effects due to surgeon-related factors. However, since presbyopia-correcting cataract surgery is typically straightforward, surgeon-related factors may not have a significant impact on the outcomes. Fourth, satisfaction results should be analyzed based on patients, not eyes, as conditions in the other eye can affect outcomes. However, some patients report satisfaction with one eye but not the other. For analyzing ocular biometric parameters as risk factors, we had to assess postoperative satisfaction per eye. Further studies should focus on patients, not individual eyes. Finally, because the follow-up period was limited to only one month for some patients, the results could have been different if these individuals had been observed for a longer and more adequate duration.

## Conclusions

In conclusion, this study suggests that the overall incidence of dissatisfaction was 18.5%, with a similar dissatisfaction rate between trifocal and EDOF IOL-implanted eyes, although the dissatisfaction types and related risk factors may differ depending on the presbyopia-correcting IOL type. Key findings indicate that preoperative myopia-related factors appear to be critical risk factors for near-vision dissatisfaction, regardless of the IOL type, whereas ocular aberrations may be related to photic disturbances depending on the IOL type. This study highlights the importance of thorough counseling, particularly for myopic patients with high ocular aberrations, before considering presbyopia-correcting IOL implantation.

## Data Availability

The datasets used and analyzed during the current study are available from the corresponding author on reasonable request.

## Abbreviations

IOL: Intraocular lenses
EDOF: Extended depth-of-focus
VA: Visual acuity
SE: Spherical equivalent
AXL: Axial length
ACD: Anterior chamber depth
CCT: Central corneal thickness
MRE: Mean refractive error
MAE: Mean absolute refractive error
HOA: Higher-order aberration
